# Arrangement of Indocyanine Green in a 1.5-Nanometer Channel to Achieve High-Efficiency Imaging of the Intestinal Lymphatic System

**DOI:** 10.3390/molecules27248704

**Published:** 2022-12-08

**Authors:** Xiangyi Kong, Nan Gao, Jianshi Du, Qing Zhao

**Affiliations:** 1 Key Laboratory of Lymphatic Surgery Jilin Province, Jilin Engineering Laboratory for Lymphatic Surgery Jilin Province, China-Japan Union Hospital of Jilin University, Changchun 130031, China; 2 Key Laboratory of Polyoxometalate and Reticular Material Chemistry of Ministry of Education, Faculty of Chemistry, Northeast Normal University, Changchun 130024, China

**Keywords:** 1.5 nanometer channel, porous organic framework, inflammatory bowel diseases, fluorescence imaging, indocyanine green

## Abstract

The complications of inflammatory bowel diseases (IBDs) seriously endanger people’s health, such as bleeding, polyp hyperplasia, and even cancer. Although the precise pathophysiology of IBD is unknown, alterations in the intestinal lymphatic network, such as lymphangiogenesis and lymphatic vessel dysfunction, are well-established features. Therefore, the development of a reliable technology is urgently required, with a stereoscopic, deep, and high-resolution technology for IBD lymphatic targeting imaging in clinical practice. However, indocyanine green, the only clinically approved imaging agent by the Food and Drug Administration, can easily cause self-aggregation or be interfered with by microenvironments, causing fluorescence quenching, which seriously affects the imaging and detective capabilities. Herein, indocyanine green molecules are arranged in a 1.5-nanometer one-dimensional channel (TpPa-1@ICG). Based on this specified structure, the fluorescence enhancement effect is observed in the TpPa-1@ICG resultant, and the fluorescence intensity is enhanced by 27%. In addition, the ICG-incorporated porous solid reveals outstanding solvent (dichloromethane, tetrahydrofuran, etc.) and thermal (>300 °C) stability. After modifying the target molecules, TpPa-1@ICG showed excellent imaging ability for intestinal lymphatic vessels, providing a new imaging tool for IBDs research.

## 1. Introduction

Inflammatory bowel diseases (IBDs), consisting of ulcerative colitis (UC) and Crohn’s disease (CD), are characterized by chronic inflammation of the gastrointestinal tract in genetically susceptible individuals exposed to environmental risk factors [1,2]. The complications of IBDs seriously endanger people’s health, such as bleeding, perforation, toxic bowel dilatation, polyp hyperplasia, or cancer. Although the precise pathophysiology of IBDs is unknown, alterations in the intestinal lymphatic network, such as lymphangiogenesis and lymphatic vessel (LV) dysfunction, are well-established features of human and experimental IBDs [1,2]. On one hand, LVs dysfunction can cause the transport substances in LVs, such as chylomicra, fatty acids, macrophages, and inflammatory factors, to enter the intestine, leading to or aggravating IBDs. On the other hand, IBDs will further stimulate lymphangiogenesis, which is mediated by binding of the lymphatic vascular endothelial selective growth factors Vascular Endothelial Growth Factor-C (VEGF-C) and VEGF-D–VEGF-R3. Anti-lymphatic treatment with anti-VEGF-R3 antibodies in an animal model of IBD has been shown to aggravate inflammation and submucosal edema, increase leukocyte infiltration, and to cause tortuous LVs. Such lymphangiogenic expansion might enhance classic intestinal lymphatic transport, eliminating excess accumulations of fluids, inflammatory cells, and mediators, and could therefore be interpreted as an “adaptive” response to acute and chronic inflammatory processes. However, whether these new LVs are functional, dysregulated, or immature is currently an area under investigation. Therefore, the imaging study of intestinal LVs is of great significance both in diagnosis and treatment.

Nowadays, CT and MRI have become the most commonly used examination techniques in clinical practice. However, it cannot be ignored that the radioactivity of CT technology increases the risk of malignant tumors, especially for young patients who undergo multiple examinations [3,4]. MRI technology is less accurate in the diagnosis and evaluation of enteritis due to severe artifacts from respiratory motion or bowel motility that obscure intestinal imaging [5,6,7]. Therefore, the development of a stereoscopic, deep, and high-resolution technology is urgently needed for enteritis imaging.

Indocyanine green (ICG) angiography is an advanced imaging technology, which can accurately and clearly image the choroidal circulation system [8]. Because the ICG molecule possesses a long wavelength with a fluorescence emission peak in the range of 795–805, it can effectively pass through pigments and hemorrhagic obstructions, realizing a clear imaging not readily achievable with other technologies. Based on these advantages, the ICG molecule became the only near-infrared dye approved by the Food and Drug Administration (FDA) for clinical in vivo application [9,10]. However, the practical application of ICG agents has also shown many shortcomings, especially in the case of high concentrations, where their self-aggregation will lead to fluorescence quenching, which seriously affects the in vivo application [11,12]. In addition, its disadvantages also include poor aqueous stability, no selectivity for lymphatic vessels and blood capillaries, and rapid clearance from lymph nodes after its administration [13,14].

Porous organic frameworks (POFs) are a new type of porous material formed by connecting organic building units with specific geometric configurations through covalent bonds [15,16,17,18,19,20]. One can precisely construct porous channels with a uniform pore size and functionalized surface by selecting the building blocks [21,22]. In this paper, we have prepared a classical porous organic framework (TpPa-1) with a uniform pore size of ~1.5 nm. On one hand, a 1.5 nm pore size is more suitable for a single ICG molecule to enter the channel. On the other hand, and more importantly, the pore walls contain a large number of carbonyl groups and Schiff-base groups that will serve as acceptors and donors of hydrogen bonds of ICG guests. Based on such a unique structure, ICG is incorporated into the interior channel of TpPa-1 in a large amount, and the separated ICG molecules effectively avoid the self-aggregation and microenvironment effects, to realize the long-term and high-intensity fluorescence of ICG. Subsequently, the ICG/TpPa-1 complex was modified with hyaluronic acid (HA) (targeting lymphatic vessel endothelial receptor-1 (LYVE-1), which is overexpressed on the lymphatic endothelium) and galactose (Gal) (targeting the intestine) (abbreviated as TpPa-1@ICG-HG) [23,24]. In this way, the TpPa-1@ICG-HG system, with strong fluorescence emission, would be an excellent intestinal lymphatic imaging agent, providing a new imaging tool for IBDs research.

## 2. Results

### 2.1. Characterization of TpPa-1@ICG

As shown in the IR spectroscopy (Figure S1 (Supplementary Materials)), several characteristic bands were observed for the ICG-adsorbed TpPa-1 sample. The formation of hydrogen bonds could be demonstrated by the blue-shift of -N-H bending from 1520 cm^−1^ (pure TpPa-1) to 1504 cm^−1^ (TpPa-1@ICG). A similar phenomenon also occurred in the -C=O stretching band with a variation from 1607 cm^−1^ (pure TpPa-1) to 1595 cm^−1^ (TpPa-1@ICG).

**Figure 1:**
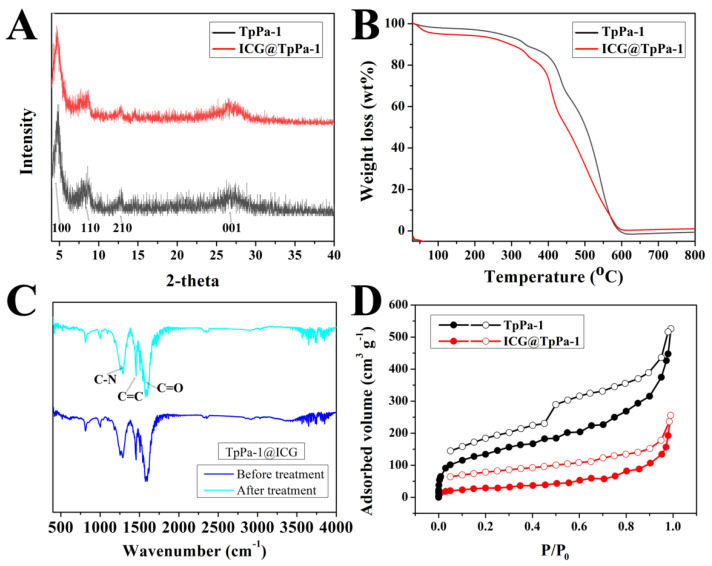
PXRD patterns (**A**) and TGA curves (**B**) of TpPa−1 and TpPa−1@ICG. FT-IR spectra (**C**) of TpPa−1@ICG before and after treatment. N_2_ adsorption–desorption isotherms (**D**) of TpPa-1 and TpPa−1@ICG.

As depicted in Figure 1A, powder X-ray diffraction (PXRD) patterns of TpPa-1 and ICG-adsorbed TpPa-1 revealed an intense peak center at 4.7°, attributed to the reflection of the (100) plane. Several minor peaks were also exhibited at 2θ = 8.3, 11.1, and 27° for both TpPa-1 and TpPa-1@ICG, proving that the TpPa-1 framework maintained its structural integrity after the incorporation of ICG molecules. As shown in Figure 1B, there was almost no weight loss before 300 °C for TpPa-1, and about 5% weight loss before 100 °C for TpPa-1@ICG, which is ascribed to the existence of solvent molecules with low boiling points. The results show that both TpPa-1 and TpPa-1@ICG owned excellent thermal stability. TpPa-1@ICG powder was not dissolved or decomposed in some solvents including dichloromethane, ethanol, acetone, chloroform, etc., after 12 h soaking experiment by using the respective solvent above (Figure 1C).

Nitrogen adsorption–desorption isotherms were recorded at 77 K to study the porosity of both TpPa-1 and TpPa-1@ICG. Calculated by using the BET model (Figure 1D), the specific surface area of TpPa-1 was 486 m^2^ g^−1^; and the value of TpPa-1@ICG was 103 m^2^ g^−1^.

The morphology of TpPa-1 solids was investigated by scanning electron microscopy (SEM), which illustrated that both TpPa-1 and TpPa-1@ICG were aggregated by micro-sized particles with an average size of 300 nm (Figure S2). Energy-dispersive X-ray spectroscopy (EDS) analysis of the C, N, O, and S elements confirmed the chemical compositions of TpPa-1 and TpPa-1@ICG, and a noticeably increased intensity for S element in TpPa-1@ICG further manifested the successful complexing of ICG molecules in the TpPa-1 network. According to elemental analysis, the ICG uptake capacity was calculated to be ~147.5 mg g^−1^ in TpPa-1@ICG (Table S1). Furthermore, after ICG molecules were incorporated in the TpPa-1 channels, the uniform-distributed ICG agent achieved rendering a ~27% increase in fluorescence intensity compared with the pristine TpPa-1 network (Figure S3).

### 2.2. Build LVs-Targeted TpPa-1@ICG

It was widely reported that lymphatic vessel endothelial receptor-1 (LYVE-1), which is overexpressed on lymphatic endothelium, and hyaluronic acid (HA) acted as the substrate for LYVE-1 [23,24]. Meanwhile, galactose (Gal) was reported to target the intestine [25]. Therefore, in order to build the intestinal LVs imaging agent, HA and Gal were chemically decorated on the surface of TpPa-1@ICG through an esterification reaction (TpPa-1@ICG-HG), which was a common and reliable strategy to render the targeted recognition.

**Figure 2:**
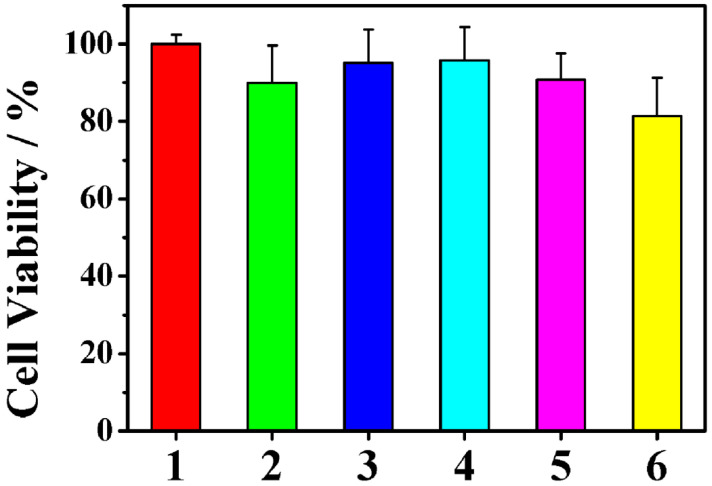
The cell toxicity of ICG, TpPa-1@ICG and different concentrations of TpPa-1@ICG-HG co-incubated with RAW cells for 24 h. Each experiment had been repeated three times. Error bars indicate ± s.d. 1: control (RAW cells only); 2: add 0.1 mg/mL ICG; 3: add 0.1 mg/mL TpPa-1@ICG; 4: add 0.1 mg/mL TpPa-1@ICG-HG; 5: add 1.0 mg/mL TpPa-1@ICG-HG; 6: add 2.0 mg/mL TpPa-1@ICG-HG.

Before the following experiments, the potential biotoxicity of TpPa-1@ICG-HG should be tested by MTT assay. The result indicated that no apparent cytotoxicity was observed, even when the TpPa-1@ICG-HG concentration was as high as 2.0 mg/mL (Figure 2). The selective fluorescence imaging properties of TpPa-1@ICG-HG were investigated in various cell types, including mouse lymphatic endothelial cells (MLEC), murine macrophage (RAW 264.7) cells, and mouse colon cancer cells (CT26).

### 2.3. Targeting LVs by TpPa-1@ICG-HA Proving by Cell Assays

**Figure 3:**
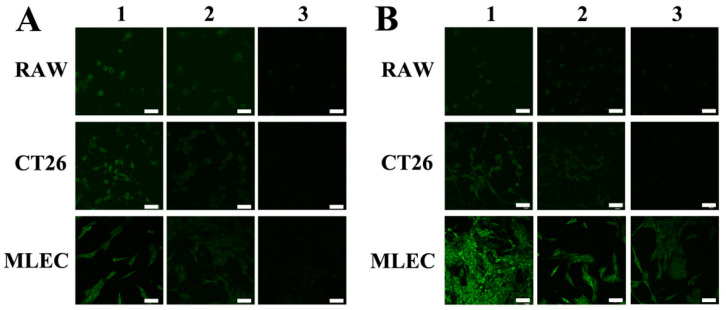
Staining effect of ICG (**A**) or TpPa-1@ICG-HG (**B**) on three kinds of cells. The numbers 1, 2, and 3 in the picture represent the cleaning of cells for 1, 2, or 3 times after staining. The scale bars equal 50 μm.

Figure 3A shows the staining effect of ICG on three kinds of cells and Figure 3B shows the staining effect on three kinds of cells using TpPa-1@ICG-HG. The numbers 1, 2, and 3 in the figure represent the cleaning of cells for one, two, and three times after staining. The results showed that, after co-incubation of ICG, the three kinds of cells were all stained, and after three rounds of flushing, the fluorescence of the cells could not be examined. In contrast, TpPa-1@ICG-HG only stained MLECs well, and after three rounds of flushing, the fluorescence of MLECs could also be observed.

### 2.4. Construction of Rat IBD Models

**Figure 4:**
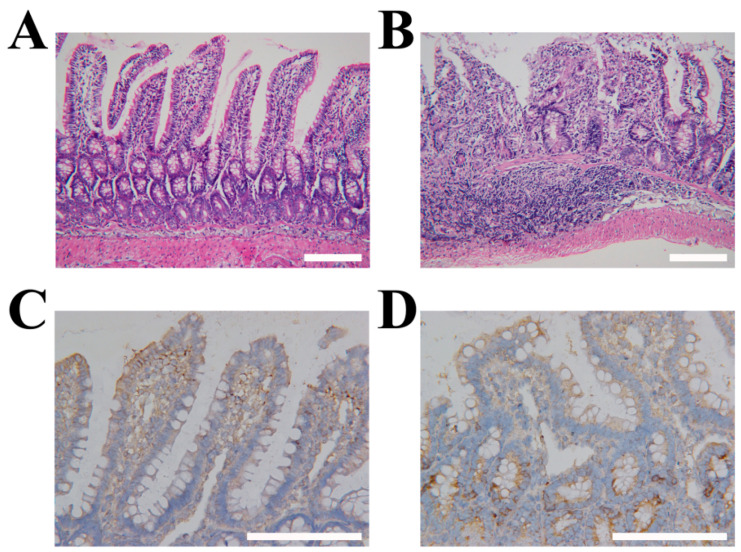
Staining rats’ intestines slices with HE or LYVE-1 antibody (the second antibody was HRP labeled). (**A**) Normal rats’ intestines slice stained with HE. (**B**) DSS- and alcohol-treated rats’ intestines slice stained with HE. (**C**) Normal rats’ intestines slice stained with LYVE-1 antibody. (**D**) DSS- and alcohol-treated rats’ intestines slice stained with LYVE-1 antibody. The scale bars equal 200 μm.

To further test the applicability of TpPa-1@ICG-HG, the rat IBD model was constructed. A total of 5 % (*w*/*v*) Dextran Sulfate Sodium salt (DSS) and 30 % alcohol (v/v) were used simultaneously to build the model [26,27,28]. Subsequently, the intestines of rats were divided into two kinds of slices. One was stained with hematoxylin–eosin (HE) staining, and the other was immunohistochemically labeled with LYVE-1 antibody (the second antibody was HRP labeled) (Figure 4). HE staining showed that the intestinal structure of rats had a regular villus structure and the intestinal wall was complete before treatment. After treatment with DSS and alcohol, the villus structure was destroyed, and the intestinal wall was no longer complete (Figure 4A,B). The results of immunohistochemistry showed that the lymphatic vessels in the rats’ intestines were evenly distributed before treatment. After treatment with DSS and alcohol, it could be seen that the lymphatic vessels proliferated and were no longer distributed evenly (brown areas in Figure 4C,D). Additionally, combined with other tests (Figures S4 and S5), the results show that the rat IBD model was successfully constructed.

### 2.5. Targeting LVs by TpPa-1@ICG-HA Proving by Rat IBD Models

**Figure 5:**
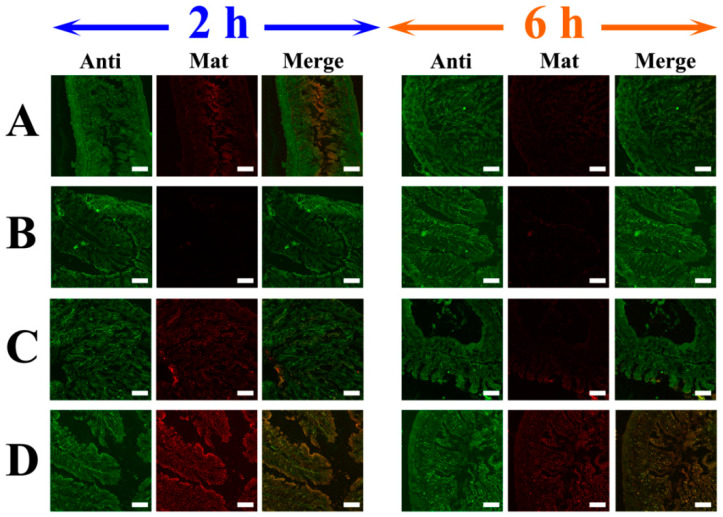
Rats’ intestines ultralow temperature slices after oral administration with ICG (**A**); TpPa-1 (**B**); TpPa-1@ICG (**C**); or TpPa-1@ICG-HG (**D**) for 2 h or 6 h. Then, the slices were further stained with LYVE-1 antibody (the second antibody was FITC labeled). The fluorescence of the antibody was displayed in green and the fluorescence of ICG, TpPa-1, TpPa-1@ICG, or TpPa-1@ICG-HG was displayed in red. Anti: antibody; Mat: material. The scale bars equal 200 μm.

Subsequently, 10 mg/kg body weight of ICG, TpPa-1, TpPa-1@ICG, or TpPa-1@ICG-HG was given by oral administration. After 2 h or 6 h, the rats were sacrificed, and the intestinal tissues were quickly taken out and placed in liquid nitrogen for cooling. Then, the intestinal tissues were sliced by ultralow temperature slicer and stained with LYVE-1 antibody directly (the second antibody was FITC labeled). The confocal microscope was used to observe the co-location of antibody/ICG or antibody/ TpPa-1@ICG-HG (Figure 5).

The results showed that, after intragastric administration for 2 h, the ICG, TpPa-1@ICG, and TpPa-1@ICG-HG could all image intestinal LVs, while TpPa-1 alone had no fluorescence. However, compared to TpPa-1@ICG-HG, the accuracy of imaging of ICG for intestinal LVs was low and the specificity to LVs is weak, shown as the poor co-location with LYVE-1 antibody. Similarly, although the fluorescence intensity of TpPa-1@ICG was stronger than ICG, TpPa-1@ICG still had poor specificity to LVs. On the contrary, the accuracy of imaging of TpPa-1@ICG-HG for intestinal LVs was high and the co-locations between LYVE-1 and TpPa-1@ICG-HG were well fitted. After intragastric administration for 6 h, ICG and TpPa-1@ICG were almost metabolized in the body, which was shown as very low fluorescence imaging intensity. In contrast, TpPa-1@ICG-HG could still image intestinal LVs clearly, which indicated that TpPa-1@ICG-HG had a good retention effect in vivo.

## 3. Discussion

**Figure 6:**
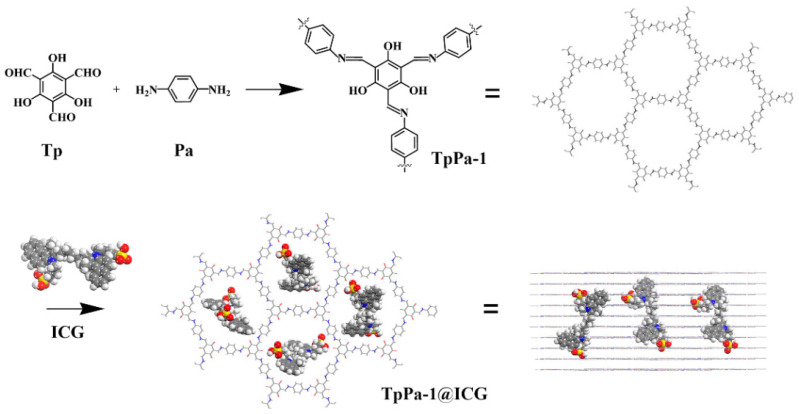
Synthesis of TpPa-1 and TpPa-1@ICG [29,30,31].

Triformylphloroglucinol (Tp) and paraphenylenediamine were adopted as building monomers to synthesize the POF network, TpPa-1, with 1.5-nanometer one-dimensional channels [29,30,31]. Due to the irreversible proton tautomerism of the enol−imine (OH) form, the keto−enamine form, the crystalline TpPa-1 possessed remarkable acid/base/thermal stability, facilitating the application in the field of biological imaging (Figure 6). Accordingly, the ICG agent was introduced into the one-dimensional POF channels through a facile immersion process, TpPa-1@ICG. Due to the confinement effects originating from the rigid POF architecture, the separated ICG molecule was successfully arranged in the ordered porous network (Figure 6).

Then, the structure of TpPa-1@ICG was characterized by several tests. The IR spectroscopy results proved the hydrogen binding of ICG molecules in the TpPa-1 network [31]; the PXRD results demonstrated the outstanding physical and chemical stability of ICG-incorporated TpPa-1, suggesting the huge potential of TpPa-1@ICG for practical application; in nitrogen adsorption–desorption isotherms’ assay, the decrease in the surface area was ascribed to the fact that ICG molecules occupied the pore space of the TpPa-1 architecture, which decreased the storage space, and resulted in the decrease in the specific surface area.

To date, ICG is the only drug approved and certified by the FDA agent for LVs imaging [32,33,34,35,36,37,38]. However, ICG molecules are easily aggregated inside the organism, which seriously suppresses the fluorescence capability and limits their stereoscopic, deep, and high-resolution imaging. Therefore, the enhanced fluorescent performance of TpPa-1@ICG, together with the outstanding thermal and solvent stability, enabled the great potential of TpPa-1@ICG in the field of biological imaging.

**Scheme 1:**
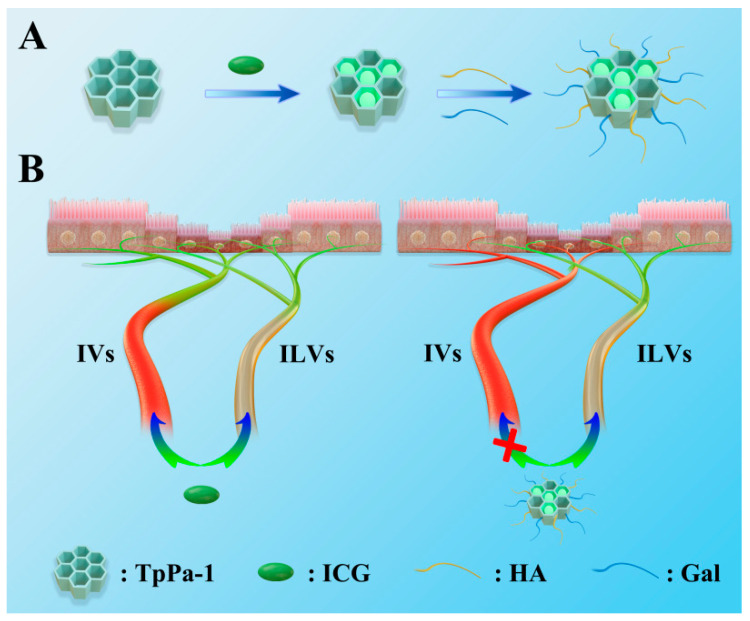
(**A**) Synthesis process of TpPa-1@ICG-HG. (**B**) Schematic of intestinal LVs targeting fluorescence imaging of TpPa-1@ICG-HG (IVs: Intestinal Vessels; I LVs: Intestinal Lymphatic Vessel).

In the selective fluorescence imaging properties of TpPa-1@ICG-HG assay, three kinds of cells were investigated. The reasons for selection were: RAW264.7 is a normal mouse cell and does not contain the LYVE-1 receptor; CT26 is a mouse cancer cell containing a small amount of the LYVE-1 receptor; and MLEC is a mouse lymphatic endothelial cell and contains a large number of LYVE-1 receptors. According to the imaging results, it can be concluded that the use of ICG staining is neither selective nor firm in binding to cells (easy to be metabolized). Meanwhile, when stained with TpPa-1@ICG-HG, both selectivity (specific binding to MLEC) and firm binding to cells (not easy to be metabolized) could be observed. In conclusion, the strong binding affinity between HA and LYVE-1 could facilitate the migration and retention of TpPa-1@ICG-HG for effective lymphatic vessel imaging. Additionally, in the rat IBD models, TpPa-1@ICG-HG was endowed with better imaging legibility and targeting than ICG. Therefore, TpPa-1@ICG-HG has the potential to become an excellent intestinal LVs imaging agent, providing a new diagnostic tool for future IBDs research (Scheme 1).

## 4. Materials and Methods

### 4.1. Chemicals

All chemicals were purchased from commercial suppliers and used as received, unless it was otherwise noted. *N*,*N*-Dimethylformamide (DMF), and triethylamine (Et_3_N) were dehydrated with Mg_2_SO_4_. Tetrahydrofuran (THF) was distilled in the presence of sodium benzophenone ketyl under an N_2_ atmosphere.

### 4.2. Synthesis of POF Sample with 1.5-Nanometer Pore Channels (TpPa-1)

A total of 63 mg Triformylphloroglucinol (Tp, 0.3 mmol) and 48 mg paraphenylenediamine (Pa-1, 0.45 mmol) were poured into a mixture of 1.5 mL of mesitylene, 1.5 mL of dioxane, and 0.5 mL of 3 M aqueous acetic acid in a Pyrex tube. Sonicated for 10 min, the system was frozen in a liquid N_2_ bath, and then degassed by three freeze–pump–thaw cycles. After being sealed off, the frozen tube was thawed at room temperature for 1 h, and then heated at 120 °C for 72 h. Finally, the red precipitate was collected by filtration and washed with anhydrous tetrahydrofuran to obtain a dark red powder POF sample, TpPa-1.

### 4.3. Synthesis of ICG Molecules Incorporated POF (TpPa-1@ICG)

A total of 55 mg of ICG powder was added to 5 mL of water, and then the ICG solution was charged into a POF water mixture containing 40 mg POF powder and 5 mL water. After stirring for 12 h, the precipitate was collected by filtration and washed with respective water, acetone, and dichloromethane for 10 min to obtain the ICG molecules incorporated POF, TpPa-1@ICG.

### 4.4. Characterization

Fourier transform infrared spectroscopy (FTIR) was conducted on the Nicolet Impact 410 (Nicolet Instrument Corporation, Madison, WI, USA). Thermogravimetric analysis (TG) was implemented using a Netzch Sta 449c thermal analyzer (Netzch, Shanghai, China) with a heating rate of 10 °C min^−1^ under air conditions. Scanning electron microscopy (SEM) imaging and energy-dispersive spectroscopy (EDS) were performed on a JEOS JSM 6700 (JEOL USA, Inc., Peabody, MA, USA). The N_2_ adsorption–desorption measurement was analyzed on a USA Quantachrome Autosorb-IQ gas adsorption analyzer (Anton Paar GmbH, Shanghai, China). Powder X-ray diffraction (XRD) measurements were analyzed on a Smartlab instrument (Rigaku Beijing Corporation, Beijing, China) with Cu Kα (λ = 1.5418 Å) radiation and X-ray 40 kV/30 mA over the angular range 2θ 4°–40° at a scan rate of 10° min^−1^.

### 4.5. Cellular Accumulation and Quantification of the Fluorescence Intensity

MLEC cells, RAW 264.7 cells, and CT26 cells (purchase from Procell Life Science & Technology Co., Ltd., Wuhan, China.) were seeded on six-well plates at 1 × 10^5^ cells per well. After 12 h incubation, cells were treated with ICG or ICG@POP. Then, cells were washed at different times with PBS and incubated in a fresh medium. The fluorescence of cells was estimated by confocal laser scanning microscope (CLSM) (FV3000, Olympus, Beijing, China).

### 4.6. Rat IBD Model Construction

The rat (purchase from Changchun Yisi Experimental Animal Technology Co., Ltd., Changchun, China) IBD model was constructed with 5% (*w*/*v*) Dextran Sulfate Sodium salt (DSS) and 30% alcohol (*v*/*v*) simultaneously. Normal drinking water was replaced by 5% DSS and rats were given 15 mL 30% alcohol per kg body weight each day by oral administration [26,27,28]. The intestinal samples were detected by HE stain and antibody stain.

Subsequently, 10 mg per kg body weight of ICG, TpPa-1, TpPa-1@ICG, or TpPa-1@ICG-HG was given by oral administration. The rats were sacrificed 2 h and 6 h after intragastric administration, and the intestinal tract was quickly removed and placed in liquid nitrogen for cooling. Subsequently, the above samples were sectioned at ultralow temperature and fluorescently stained with LYVE-1 antibody (the second antibody was FITC labeled).

### 4.7. Rat Intestinal Slices Assays

After the rat IBD model was constructed, the intestines of rats were longitudinally incised and cleaned with physiological saline three times. Then, the intestinal tissues were embedded with paraffin and sliced. A part of the sections was stained with hematoxylin–eosin (HE) staining, and other sections were immunohistochemically labeled with LYVE-1 antibody (the second antibody was HRP labeled).

For the ultralow temperature section, after rats were sacrificed, the intestinal tissues were quickly taken out and placed in liquid nitrogen for cooling. Then, the tissues were sliced with an ultralow temperature slicer and direct stained with LYVE-1 antibody (the second antibody was FITC labeled).

## 5. Conclusions

The complications of IBDs seriously endanger people’s health, therefore it is urgently needed to develop a reliable technology with a stereoscopic, deep, and high-resolution technology for IBD lymphatic targeting imaging in clinical practice. However, the FDA-approved imaging agent, ICG, can easily cause self-aggregation or be interfered with by microenvironments, causing fluorescence quenching, which seriously affects the imaging and detective capabilities. In order to improve the imaging capability of ICG, TpPa-1 was designed and synthesized, which, with 1.5 nanometer 1D channels, was adopted as a novel container to incorporate the ICG molecules to obtain a high-performance imaging agent. Due to the uniform arrangement, the resulting solid achieved a fluorescence enhancement with a coefficient of 27% and outstanding thermal/solvent stability. After modifying the target molecules, TpPa-1@ICG-HG showed excellent imaging ability for both MLEC cell and intestinal lymphatic vessels, providing a new diagnostic tool for future IBDs’ research.

## Data Availability

All data related to this study are presented in this publication.

## Supplementary Materials

The following supporting information can be downloaded at: https://www.mdpi.com/article/10.3390/molecules27248704/s1, Figure S1: FT-IR spectra of ICG, TpPa-1, and TpPa-1@ICG (a) and the enlarged spectra (b); Figure S2: SEM images for TpPa-1 (a) and TpPa-1@ICG (b); Figure S3: Fluorescence emission spectra of ICG (black) and TpPa-1@ICG (red) dispersed in water (0.25 mg mL^−1^); Figure S4: Change in body weight obtained from rat oral administration of DSS or alcohol. Control groups mean no administration; Figure S5: Hematochezia in rats after DSS and alcohol treatment. (Left) control group; (Right) DSS and alcohol treatment; Table S1: CHN element analysis.

## Sample Availability

Samples of the compounds TpPa-1, TpPa-1@ICG and TpPa-1@ICG-HG are available from the authors.
