# Supplementary material for: Arrangement of Indocyanine Green in a 1.5-Nanometer Channel to Achieve High-Efficiency Imaging of the Intestinal Lymphatic System
Source: Molecules. 2022 Dec 8;27(24):8704. doi: 10.3390/molecules27248704 (PMC9786184; doi:10.3390/molecules27248704)
Supplement: Supplementary file 1 [file molecules-27-08704-s001.zip › molecules-2070075-supplementary.pdf]

Arrangement of Indocyanine Green in a 1.5-Nanometer Channel to Achieve High-Efficiency Imaging of the Intestinal Lymphatic System

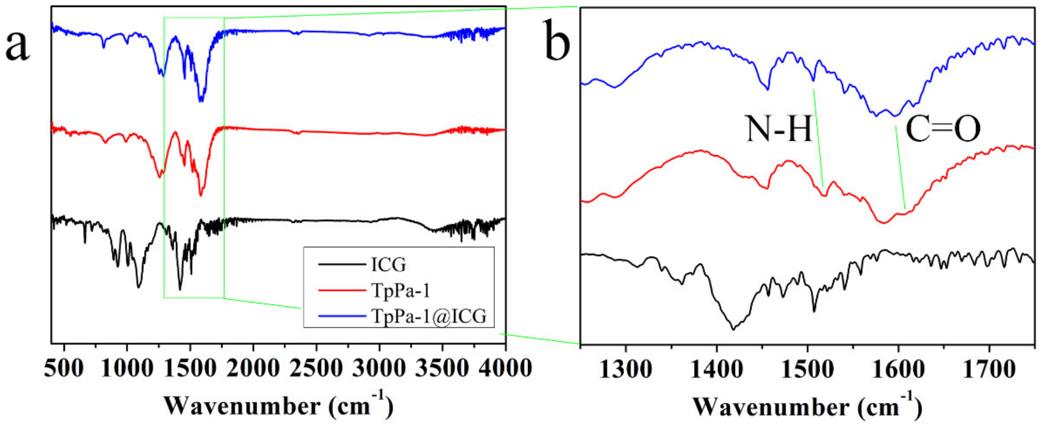

Figure S1. FT-IR spectra of ICG, TpPa-1, and TpPa-1@ICG (**a**) and the enlarged spectra (**b**).

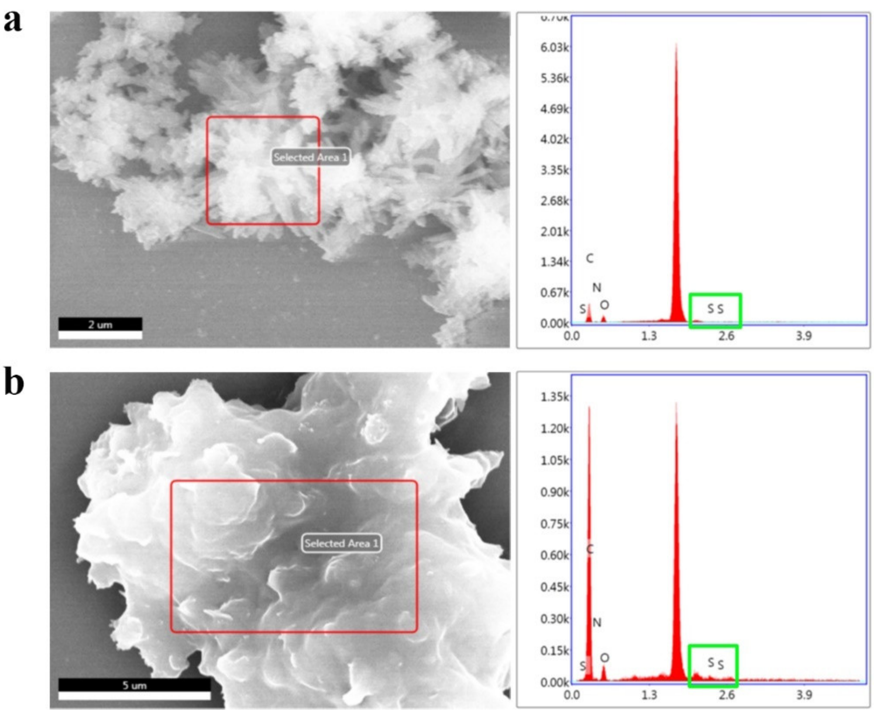

Figure S2. SEM images for TpPa-1 (**a**) and TpPa-1@ICG (**b**).

Table S1. CHN element analysis.

		C (%)	N (%)	S (%)
TpPa-1	Theoretical	67.925	13.208	~
	Experimental	65.566	14.551	~
TpPa-1@ICG	Theoretical	65.642	13.142	1.054
	Experimental	66.018	12.973	1.057

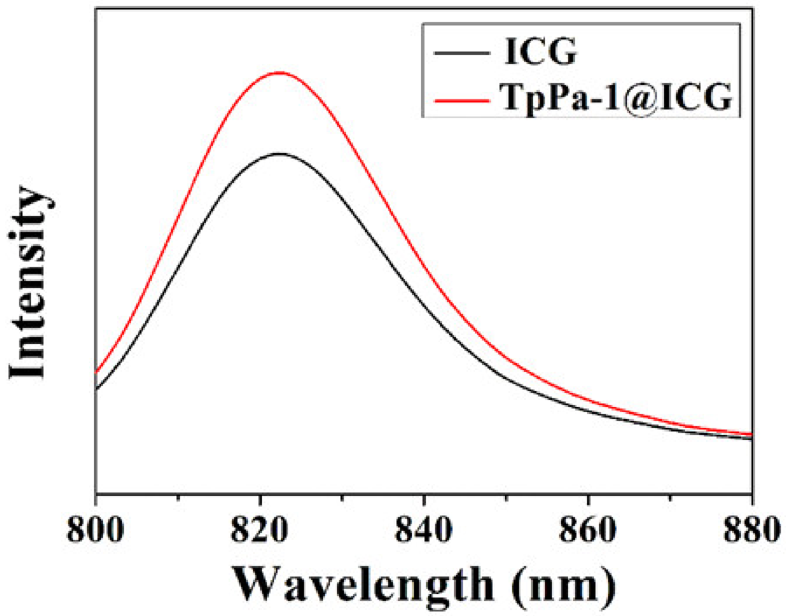

Figure S3. Fluorescence emission spectra of ICG (black) and TpPa-1@ICG (red) dispersed in water (0.25 mg mL^{-1}).

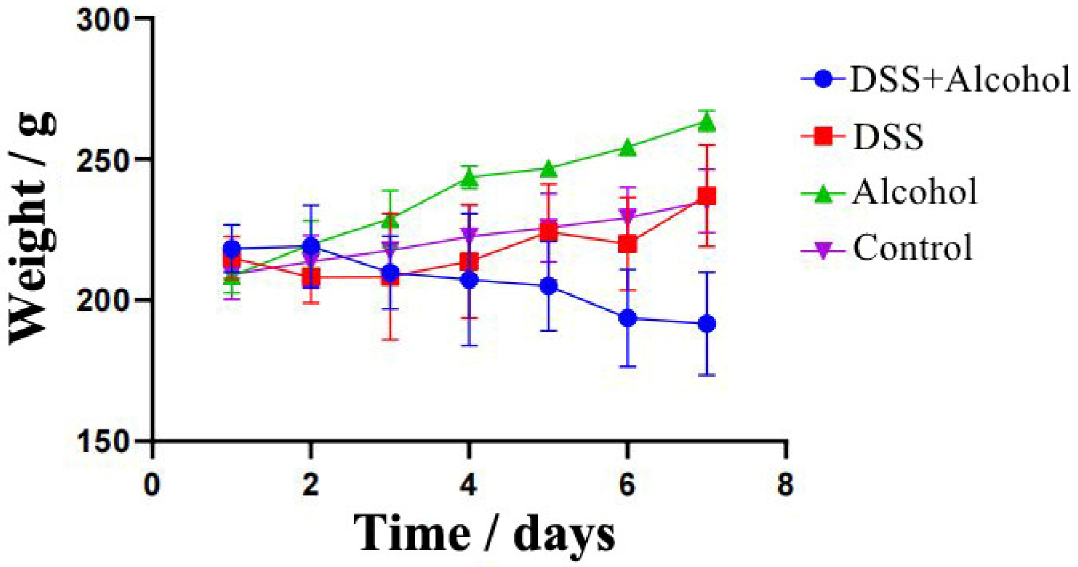

Figure S4. Change in body weight obtained from rat oral administration of DSS or alcohol. Control groups mean no administration.

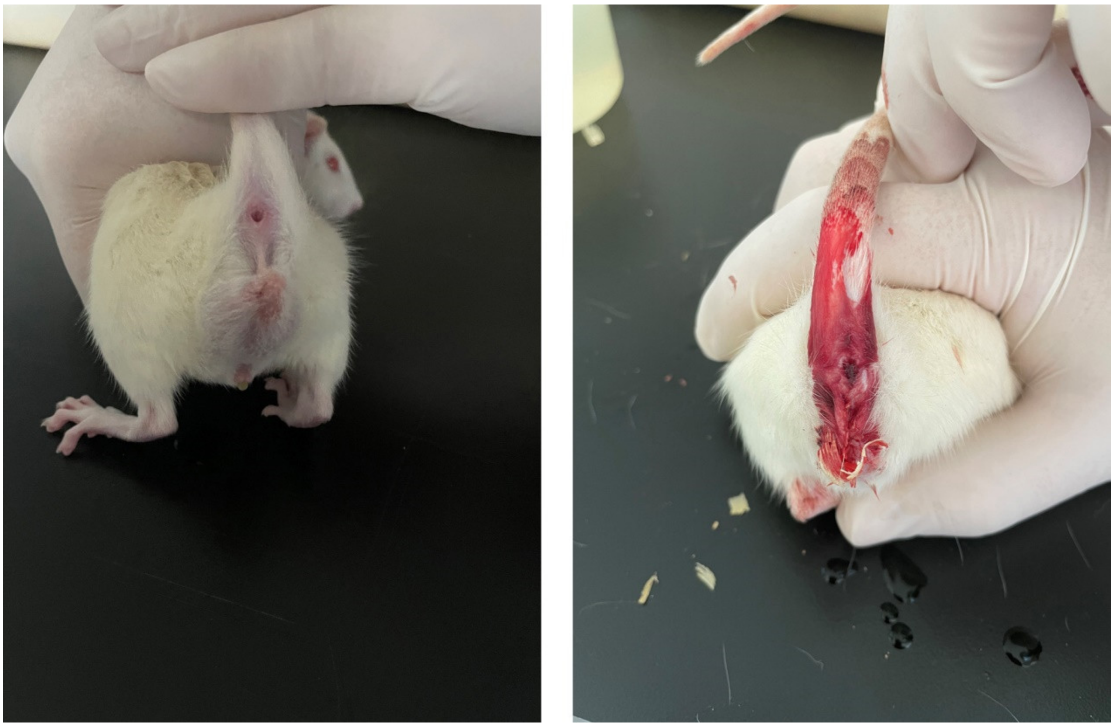

Figure S5. Hematochezia in rats after DSS and alcohol treatment. (left) control group; (right) DSS and alcohol treatment.
